# Familial Hyperaldosteronism Type IV (FH-IV)—Clinical Phenotypes, Genetics and Management of *CACNA1H*-Related Primary Aldosteronism: A Systematic Review

**DOI:** 10.3390/jcm15103693

**Published:** 2026-05-11

**Authors:** Wojciech Michalski, Igor Jaszczyszyn, Weronika Bielska, Artur Stolarczyk

**Affiliations:** 1 Department of Orthopaedics and Rehabilitation, Medical and Dentistry Faculty, Medical University of Warsaw, 02-091 Warsaw, Poland; wojciech.jan.michalski@gmail.com (W.M.); artur.stolarczyk@wum.edu.pl (A.S.); 2 Doctoral School, Medical University of Warsaw, 02-091 Warsaw, Poland; 3 Faculty of Medicine, Medical University of Łódź, 90-419 Łódź, Poland; weronika.bielska@stud.umed.lodz.pl

**Keywords:** primary aldosteronism, hyperaldosteronism, familial hyperaldosteronism type IV (FH-IV), *CACNA1H*, systematic review, genetic screening, calcium channel blockers (CCBs), mineralocorticoid receptor antagonists (MRAs)

## Abstract

**Background/Objectives**: Familial hyperaldosteronism type IV (FH-IV) is an extremely rare, clinically heterogeneous condition representing the least characterized familial subtype of primary aldosteronism (PA) caused by germline gain-of-function *CACNA1H* mutations. Despite growing molecular insights, optimal diagnostic and therapeutic strategies remain poorly defined. This systematic review aims to synthesize available evidence regarding the clinical, biochemical, and genetic characteristics of FH-IV, and to evaluate the efficacy of current pharmacological and surgical treatments. **Methods**: A systematic review was conducted in accordance with PRISMA guidelines and preregistered in PROSPERO (CRD420261324945). A comprehensive search of MEDLINE, Embase, and Web of Science identified studies reporting genetically confirmed FH-IV patients. Data concerning clinical phenotypes, diagnostic evaluations, treatment outcomes, and genetic backgrounds were extracted and analyzed. **Results**: The primary cohort included 31 fully characterized symptomatic patients, alongside 8 mutation-positive relatives (4 asymptomatic carriers and 4 symptomatic individuals). The genetic landscape was remarkably heterogeneous, encompassing 17 distinct *CACNA1H* mutations. Clinically, diagnosis was frequently delayed, often complicated by atypical normokalaemic presentations and misleading adrenal imaging. Surgical treatment was generally ineffective, frequently resulting in persistent or recurrent hypertension and biochemical dysregulation. Pharmacologically, patients often required multiple antihypertensive drugs, most frequently a combination of mineralocorticoid receptor antagonists (MRAs) and calcium channel blockers (CCBs). **Conclusions**: FH-IV is best conceptualized as a systemic adrenal channelopathy. While standard screening parameters are usually elevated, atypical biochemical profiles and misleading structural imaging can complicate the diagnostic process. Optimal management relies on multigene Next-Generation Sequencing (NGS) panels for definitive diagnosis and cascade screening of relatives. Finally, while the combination of MRAs and CCBs is commonly used in PA, it represents a valuable therapy for FH-IV, with dual L-/T-type CCBs emerging as a potential disease-specific option.

## 1. Introduction

### 1.1. Aldosterone and Its Biological Function

Aldosterone, the primary mineralocorticoid, plays a central role in maintaining homeostasis. Produced in the *zona glomerulosa* (ZG) of the adrenal cortex, this hormone exerts its physiological effects through the mineralocorticoid receptor (MR), expressed in multiple tissues. The primary physiological actions of aldosterone include sodium and water reabsorption, along with potassium and hydrogen ion secretion in the aldosterone-sensitive distal nephron [1]. The aldosterone biosynthesis is tightly regulated by two main factors: angiotensin II and potassium levels. By regulating renal ion handling, aldosterone controls blood pressure (BP), plasma volume and acid-base equilibrium, making this hormone essential for cardiovascular and renal homeostasis. Overproduction can disrupt this balance, leading to severe complications [2].

### 1.2. Primary Aldosteronism

Primary aldosteronism (PA) can be defined as excessive autonomous production of aldosterone, which results in low concentration of plasma renin, subsequent hypokalemia, and hypertension (HT) [3]. It is now a leading cause of secondary HT [4]. In patients with resistant HT, the prevalence is even higher, estimated to reach up to 20% [5]. Despite this prevalence, PA remains underdiagnosed, with screening rates frequently reported about 1–2% in eligible populations [4,6].

Recent clinical and histopathological advances redefine aldosterone excess as a broad pathophysiological continuum [7]. According to the international HISTALDO consensus, adrenal morphology in PA encompasses a diverse spectrum of lesions, ranging from aldosterone-producing micronodules (APMs) to macroscopic nodules and classical adenomas [8]. Consequently, clinical management relies primarily on functional lateralization to determine whether cases are pharmacologically or surgically curable [7,8].

Somatic mutations in ion channels and pumps of ZG cells drive the pathogenesis in the majority of sporadic cases (>90%) [6,9]; however, some patients with PA have germline mutations, leading to familial subtypes of the disease [10].

### 1.3. Consequences of Hyperaldosteronism

The pathological effects of aldosterone are mediated through both genomic (transcriptional) and non-genomic signaling pathways activated by MR engagement. Beyond its classical renal effects, aldosterone activates MR in extrarenal tissues including the heart, vessels, and brain [2].

Aldosterone excess mediates significant cardiovascular and renal damage. At the vascular level, it induces endothelial dysfunction, enhances atherosclerotic plaque development, its instability and also predisposes to higher arterial stiffness and calcification. At the cardiac level, aldosterone excess induces inflammation, fibrosis, and myocardial hypertrophy, ultimately culminating in heart failure [11]. As a result, patients with PA face a markedly elevated cardiovascular risk that extends beyond the hemodynamic effects of high BP [12]. Compared to patients with primary HT matched for age, sex, and BP, those with PA exhibit a significantly higher incidence of major cardio- and cerebrovascular events. Therefore, early diagnosis and targeted intervention are crucial to mitigate these complications [5].

### 1.4. Familial Hyperaldosteronism

**Figure 1:**
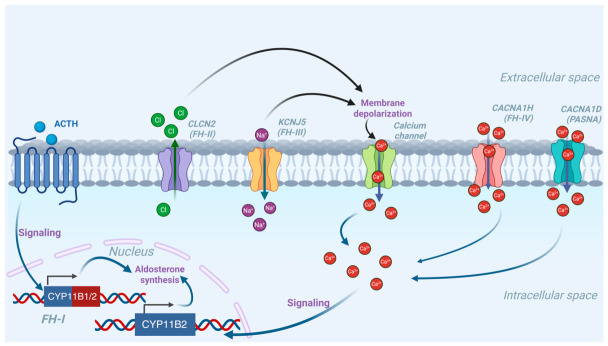
Schematic representation of molecular mechanisms underlying Familial Hyperaldosteronism (FH). Increased intracellular calcium signaling upregulates *CYP11B2*, which encodes aldosterone synthase, the essential enzyme for aldosterone biosynthesis ultimately driving its excessive production. The *CYP11B1/2* chimeric gene is directly regulated by adrenocorticotropic hormone (ACTH). Abbreviations: FH-(I–IV), Familial Hyperaldosteronism type (I–IV), PASNA, Primary Aldosteronism, Seizures, and Neurologic Abnormalities Syndrome. Created in Biorender. Michalski, W. (2026) https://biorender.com/7oiulx1 (accessed on 13 March 2026).

Familial hyperaldosteronism (FH) is a heterogeneous group of autosomal dominant disorders caused by germline mutations. Recent registry data suggest that its actual prevalence is about 1% of PA cases [13,14], though it may be underdiagnosed due to incomplete penetrance or de novo variants [9,14]. Currently distinct subtypes of FH have been characterized, each driven by unique molecular mechanisms [15] (Figure 1).

### 1.5. Diagnosis of PA and Familial Forms

PA diagnosis uses a three-step approach: screening, confirmatory testing, and subtype classification [5]. The aldosterone-to-renin ratio (ARR) is the primary screening tool, measuring plasma aldosterone concentration (PAC) and plasma renin activity (PRA) or plasma renin concentration (PRC). Current guidelines recommend screening all hypertensive individuals rather than restricting screening to resistant HT or other high-risk subgroups [16].

After a positive screen, confirmatory testing using saline infusion test (SIT), captopril challenge test (CCT) or dietary sodium suppression helps to exclude false positives [17]. However, these tests have acknowledged weaknesses and do not always provide a reliable distinction between autonomic aldosterone excess and normal physiology [16,18]. Subtype classification via adrenal computed tomography (CT) and adrenal venous sampling (AVS) distinguishes unilateral disease from bilateral disease [16].

For familial PA, diagnosis requires biochemical screening with clinical suspicion such as early-onset HT, significant family history of HT or related complications. Genetic testing is advisable in such cases. Whole exome sequencing (WES) or multigene panels examining *CACNA1H*, *KCNJ5*, *CLCN2*, *CACNA1D*, and *CYP11B1/CYP11B2* identify germline mutations [13]. Cascade genetic screening of at-risk relatives enables early intervention [15].

### 1.6. CACNA1H Encodes Calcium Channel Subunit

**Figure 2:**
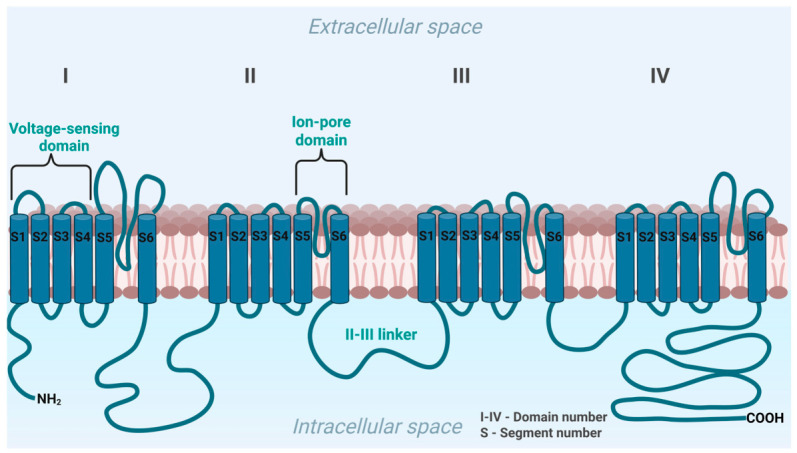
Structure of the Cav3.2 T-type calcium channel encoded by *CACNA1H*. Created in Biorender. Michalski, W. (2026) https://biorender.com/xbve32l (accessed on 13 March 2026).

The *CACNA1H* gene is located on chromosome 16 and encodes the pore-forming α_1_H subunit of the low-voltage-activated T-type calcium channel Cav3.2 [19]. Its structure is shown in Figure 2. This channel comprises 2353 amino acids organized into four homologous domains (I–IV), each containing six transmembrane helices (S1–S6). The S1–S4 segments of each repeat form the voltage-sensing domain, with the S4 segment containing positively charged residues critical for voltage sensitivity. The pore of the channel is formed by the P-loop and S5-S6 segments from each domain, allowing selective calcium entry [20]. The four domains are connected by three intracellular loops (linkers) and are flanked by cytoplasmic N- and C-termini. These intracellular regions are crucial for channel regulation and protein interactions. The II-III linker serves as a major target for phosphorylation and posttranslational modifications to modulate channel open probability, whereas C-terminus critically regulates fast channel inactivation [20,21].

Physiologically, Cav3.2 has an activation threshold of approximately −70 mV, which closely aligns with the highly negative resting membrane potential of ZG cells (~−80 mV) [22,23]. The overlap between its activation curve and its rapid voltage-dependent inactivation creates a steady-state “window current” roughly between −65 and −40 mV, uniquely allowing for continuous calcium entry at physiological resting potentials [20]. Physiological stimuli induce membrane depolarization, triggering calcium influx through these channels. This continuous calcium entry drives intracellular calcium oscillations that provide the essential second messenger signal for upregulating aldosterone synthase (*CYP11B2*) expression and driving aldosterone biosynthesis [22,24].

### 1.7. Pathophysiology of FH-IV

FH-IV is an autosomal dominant disorder caused by heterozygous germline gain-of-function mutations in the *CACNA1H* gene [21]. The classic and most frequently identified mutations, such as M1549V and M1549I, are located in the highly conserved S6 segment of repeat III, a region vital for rapid channel inactivation. These mutations profoundly alter the channel’s biophysical properties by causing drastically delayed and impaired inactivation, alongside a significant leftward shift in the activation curve to more hyperpolarized potentials. Consequently, the mutant channels activate at voltages closer to the resting membrane potential, exhibit an enlarged window current, and remain open significantly longer [19,21]. In vivo studies using *CACNA1H* knock-in mouse models (M1560V/+) demonstrate that this defect directly leads to increased baseline and peak intracellular calcium concentrations in ZG cells. This chronic intracellular calcium overload serves as a constitutive signal that drives autonomous *CYP11B2* transcription and excessive aldosterone production, remarkably without increasing the actual frequency of calcium spikes [25].

Interestingly, these point mutations exert splice-variant-specific effects, causing profound gain-of-function changes specifically in the long isoform of Cav3.2 (+exon 26) that is exclusively expressed in human ZG cells. This tissue-specific alternative splicing helps explain why the clinical phenotype is generally restricted to PA without broader neurological manifestations [26]. Nevertheless, there is significant phenotypic diversity among patients [19,20].

### 1.8. Rationale

Despite the growing literature and significant advances in the genetic, clinical, and therapeutic understanding of PA, including its familial forms, FH-IV remains the least characterized subtype [13]. Existing clinical data are limited mostly to isolated case reports, small case series, and functional in vitro or animal studies [15]. The rarity of FH-IV makes it challenging for clinicians to properly diagnose patients with this form of PA. Furthermore, the phenotypic spectrum of FH-IV appears broader than initially recognized, ranging from severe early-onset HT to normotensive presentations with isolated hypokalemia [27]. This leads to prolonged periods of unmanaged aldosterone excess. Moreover, the therapeutic management of FH-IV presents a specific clinical challenge. While standard pharmacological treatment of FH relies on MRAs [15], the pathophysiology of FH-IV suggests that other treatments may be warranted [20,28]. A systematic review is essential for integrating existing data, reducing uncertainty in clinical decision-making, and guiding future diagnostic protocols.

### 1.9. Objectives

Consequently, the primary objective of this systematic review is to synthesize available evidence regarding the clinical, biochemical, and genetic characteristics of FH-IV.

The specific objectives are:

1. To define the clinical phenotype of FH-IV, specifically analyzing the age of onset, severity of HT, and prevalence of hypokalemia among *CACNA1H* mutation carriers.
2. To evaluate genotype-phenotype correlations, assessing whether specific amino acid substitutions within the Cav3.2 channel are associated with more severe clinical presentations.
3. To characterize the findings of standard adrenal imaging and venous sampling to determine their utility in distinguishing FH-IV from sporadic forms.
4. To determine the efficacy of therapeutic interventions, comparing clinical outcomes between patients treated with MRAs, CCBs, and surgical adrenalectomy.

## 2. Materials and Methods

### 2.1. Review Methodology

This systematic review was conducted in accordance with the Preferred Reporting Items for Systematic Reviews and Meta-Analyses (PRISMA) guidelines [29]. The PRISMA 2020 checklist is provided in Supplementary Material (Table S1). The review protocol was developed prior to study initiation, and the research question was structured according to the Population, Intervention, Comparison and Outcome (PICO) framework [30]. The study protocol was prospectively registered in the PROSPERO database (registration number: CRD420261324945).

### 2.2. Searching Criteria

A comprehensive literature search was conducted in the following electronic databases: PubMed/MEDLINE, Embase, and Web of Science. No time or language restrictions were applied. The final search was performed in February 2026. The search strategy combined Medical Subject Headings (MeSH) and free-text terms related to FH-IV. Boolean operators (AND, OR) were used to combine keywords. The search strategy was adapted to the specific requirements of each database. All retrieved records were imported into RAYYAN-QCRI software [31], where duplicates were automatically identified and subsequently manually verified by W.M. Any duplicate records were removed. Screening was independently conducted in blinded mode by W.M. and I.J. The selection process consisted of two stages: title and abstract screening followed by full-text assessment. In cases of unresolved disagreement, a third reviewer (W.B.) was consulted. The reference lists of all included studies were manually screened to identify additional relevant publications. All eligible studies were subsequently imported into Zotero reference management software [32]. The complete search strategy is provided in Supplementary Material (Supplementary S1) and in the PROSPERO registration record.

### 2.3. Eligibility Criteria

The inclusion and exclusion criteria were predefined by the authors and established through consensus-based discussion. These criteria were developed to ensure methodological consistency and relevance to the research question. All eligibility criteria applied during the study selection process are summarized in Table 1.

### 2.4. Data Extraction, Correction and Synthesis

Data were extracted independently by W.M. and W.B., with an additional verification performed by I.J., using a predefined Excel data extraction sheet (Microsoft Corporation, Redmond, WA, USA). Data extraction was conducted according to predefined categories relevant to the diagnosis and management of FH. The main categories included: patient characteristics, laboratory findings, imaging diagnostics, treatment strategies and clinical outcomes, family history, and genetic background. The specific variables collected within each category are presented in Section 3 and Section 4. All extracted parameters were established prior to data collection through a consensus-based discussion among all authors to ensure methodological consistency and to minimize the risk of selective reporting bias. Parameters initially considered for inclusion (e.g., body mass index (BMI), urine potassium, and urinary aldosterone) were ultimately excluded from the analysis because they were not reported for the majority of patients in the included studies.

During the preparation of the patient results tables, substantial heterogeneity was observed across the included studies in terms of data availability and reporting formats. To improve data transparency, and facilitate meaningful comparisons, all clinical and biochemical parameters (including BP, potassium levels, aldosterone, renin, and ARR) were standardized to uniform units. The detailed methodology regarding specific data extraction rules, unit conversion formulas is provided in the Supplementary Material (Supplementary S2).

In the results tables, values exceeding the upper limit of normal are marked with an upward arrow (↑), values below the lower limit with a downward arrow (↓), and results within the reported reference range with a bidirectional arrow (↔). It should be noted that while most authors provided their specific laboratory reference ranges, in some cases, the original papers only offered a descriptive clinical statement of “hypokalemia” without specifying the normative values. Therefore, the downward arrows (↓) indicating hypokalemia strictly reflect the specific definitions and reference ranges provided by the authors of each original study.

### 2.5. Critical Appraisal

The methodological quality of the included studies was assessed using the Joanna Briggs Institute (JBI) Critical Appraisal Checklist for Case Series or the JBI Critical Appraisal Checklist for Case Reports, depending on the study design [33]. The checklist for case series consists of ten criteria, whereas the checklist for case reports includes eight criteria, both designed to evaluate methodological rigor and potential risk of bias. Each criterion was rated as “Yes” (Y), “No” (N), or “Unclear” (U), with a higher number of “Yes” responses indicating better methodological quality and a lower risk of bias. Quality assessment was performed independently by W.M. and I.J., and any discrepancies were resolved through discussion. For each study, a total quality score was calculated based on the number of “Yes” responses.

## 3. Results

### 3.1. Study Selection Process and Quality Assessment Results

**Figure 3:**
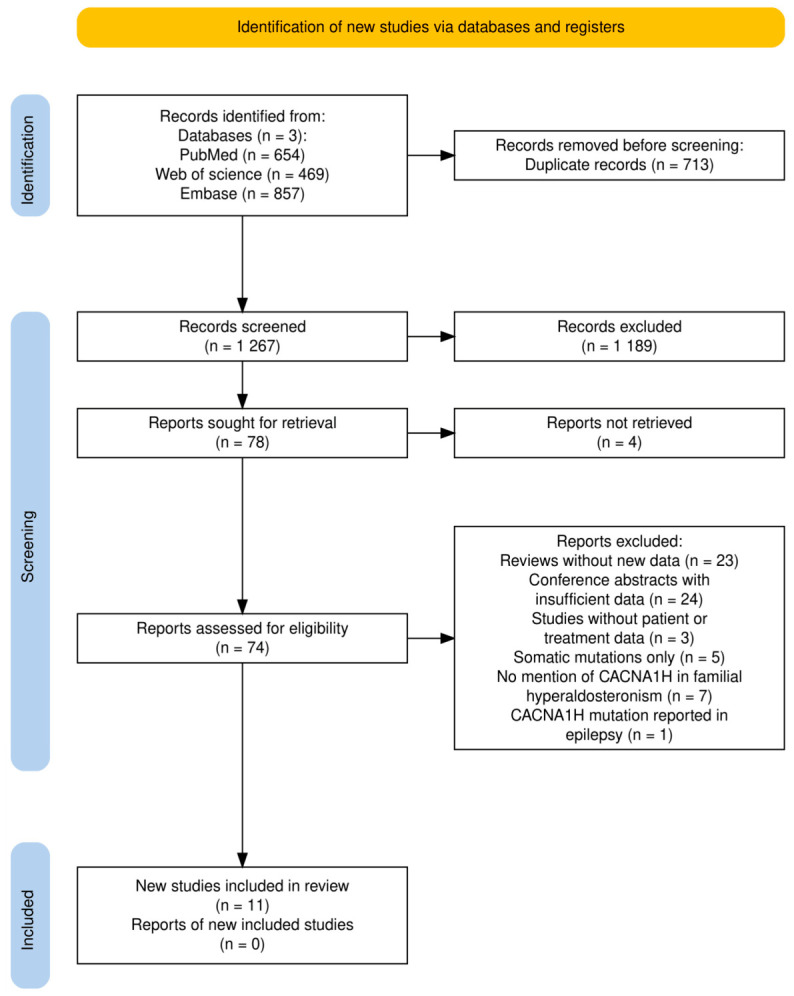
Preferred Reporting Items for Systematic Reviews and Meta-Analyses (PRISMA) flow diagram of the study selection process. Created in [43] by I.J.

The search strategy yielded a total of 1980 records. After the removal of duplicates, 1267 studies remained for screening. Following title and abstract screening, 78 articles were considered potentially eligible. Of these, 74 full-text articles were retrieved and assessed for eligibility. Ultimately, 11 studies were included in the qualitative synthesis [19,21,34,35,36,37,38,39,40,41,42], from which relevant data and outcomes were extracted. The study selection process is illustrated in the PRISMA flow diagram [43] (Figure 3). Consequently, the comprehensive clinical, laboratory, genetic, and pharmacological data extracted from the 31 main cases were summarized in detail in the subsequent tables (Table 2, Table 3, Table 4 and Table 5) and in Supplementary Material (Table S2), while data from the 8 relatives were analyzed separately to evaluate familial phenotypic variability and disease penetrance (Table 6 and Table 7).

The methodological quality of the included studies was assessed, with detailed results provided in the Supplementary Material (Supplementary S3 and S4). Among the case series, total scores ranged from 6 to 7 out of 10, while case reports scored between 5 and 7 out of 8. Most studies clearly described patient demographics, clinical presentation, diagnostic procedures, and interventions; however, reporting of follow-up outcomes, adverse events, and site-specific demographic information was occasionally incomplete or unclear.

### 3.2. Study and Patients Characteristics

Across selected studies, a total of 39 individuals carrying germline *CACNA1H* mutations were identified. To ensure rigorous quantitative evaluation, the cohort was methodologically stratified. The primary analysis focused on 31 fully clinically characterized, symptomatic patients carrying germline *CACNA1H* variants associated with FH-IV. The remaining 8 mutation-positive relatives were excluded from the main quantitative cohort to maintain data integrity. Specifically, 4 of these family members exhibited distinct clinical signs of the disease (e.g., severe HT or hypokalemia) but were excluded from the primary quantitative cohort because they either lacked the sufficient, standardized laboratory data required for a comprehensive quantitative synthesis or a formal clinical diagnosis of PA was explicitly rejected by the original investigators based on their strict biochemical criteria [19,34,42]. Furthermore, 4 genetically confirmed relatives were completely asymptomatic, normotensive carriers [19,34,39]. Including them would artificially skew the baseline clinical and biochemical characteristics of the symptomatic cohort.

The demographic profile of the primary cohort (n = 31) comprised 17 males (54.8%) and 14 females (45.2%). The age at which PA was definitively diagnosed ranged from 2 months to 75 years, with a median age at diagnosis of 36 years (mean: 34 years). The age at HT onset ranged from early infancy (2 months) to 48 years, with a median onset age of 32 years (mean: 27 years). A direct case-by-case comparison of paired data revealed a significant diagnostic delay: the median time elapsed between the initial onset of HT and the definitive diagnosis of PA was 4 years (mean: 7 years). Alarmingly, in extreme cases, the underlying syndrome remained unrecognized for up to 30 years (e.g., Patient 15 and 20) [37,39]. The reported ethnic background was globally diverse [19,21,35,36]; however, it is noteworthy that almost all subsequent cases reported since 2024 originated from Chinese medical centers [34,37,38,39,40,41,42].

The clinical presentation of the primary cohort was remarkably heterogeneous. BP at the time of initial evaluation showed significant variation; peak systolic blood pressure (SBP) ranged from 108 to 240 mmHg with a median of 160 mmHg (mean: 169 mmHg), while peak diastolic blood pressure (DBP) ranged from 70 to 160 mmHg with a median of 100 mmHg (mean: 104 mmHg). Patients presenting in infancy or early childhood frequently exhibited atypical clinical manifestations, including pyloric stenosis, significant developmental delays, and early left ventricular hypertrophy [19,21]. Adolescent and adult patients typically presented with symptoms directly secondary to long-standing HT and severe hypokalemia. Furthermore, a substantial proportion of these individuals exhibited significant metabolic and cardiovascular comorbidities, notably including structural cardiac abnormalities and severe cerebrovascular events [37,39,40,42]. A case-by-case summary of the specific clinical manifestations and co-existing conditions, as available from the original reports, is provided in Table 2.

**Table 2:** Patient characteristics.

Study [Ref.]	Pt No.	Sex	Ethnicity	Age at HT Onset [Years]	Age at PA Diagnosis [Years]	Highest BP (SBP/DBP) [mmHg]	Clinical Symptoms and Comorbidities
Scholl et al. [19]	1	M	European	3	3	160/105	Developmentally delayed
	2	F	African American	7	7	150/90	Asthma, seasonal allergies, headache, blurry vision
	3	F	NR	17	29	215/115	NR
	4	M	European	8	9	140/90	Pyloric stenosis (at 5 weeks), attention deficit disorder
	5	M	European	9	9	192/144	Headaches, enuresis, polyuria, polydipsia, left inguinal hernia, orchiopexy
	6	M	Hispanic	2 months	2 months	170/100	Left ventricular hypertrophy
Daniil et al. [21]	7	M	European	2 months	2 months	110/70	Pyloric stenosis (at 2 months), multiplex developmental disorder (at 10 years)
	8	M	French West Indies	36	51	155/95	NR
	9	F	French West Indies	32	37	153/97	NR
	10	M	West Indies	34	44	154/104	NR
	11	M	West Indies	37	39	160/98	Obesity
	12	F	European	48	50	142/87	NR
Wulczyn et al. [35]	13	F	Ashkenazi Jewish	NR	31	133/95	Ehlers-Danlos syndrome (hypermobile), POTS, extreme weakness
Liu et al. [36]	14 *	M	Chinese	14	17	150/100	Occasional headache, palpitations, chest pain upon exertion
Zhang et al. [37]	15	F	NR	31	61	220/100	Recurrent dizziness, type 2 diabetes, hyperlipidemia
	16	M	NR	33	38	220/160	Recurrent dizziness, hyperuricemia
	17	M	NR	32	36	160/110	Epistaxis, thyroid nodules, liver insufficiency, hyperuricemia
Hasini et al. [38]	18	F	NR	NR	27	108/74	Circumoral tingling and numbness, difficulty in swallowing, opisthotonos, stiffness of muscles
Zhu et al. [39]	19	M	NR	34	39	185/120	Episodes of dizziness and headache, total body paralysis due to cold exposure
	20	F	NR	45	75	212/87	Cerebral hemorrhage, 3 acute cerebral infarctions, diabetes, co-existing subclinical Cushing Syndrome
	21	M	NR	36	47	180/100	Acute cerebral hemorrhage, diabetes
	22	M	NR	36	36	164/113	Mild OSA, severe nocturnal hypoxemia
Li et al. [34]	23	F	NR	13	14	150/100	Depressive state
	24	F	NR	44	47	157/85	NR
	25	F	NR	45	54	155/93	NR
	26	F	NR	23	32	160/100	Type 2 diabetes, intellectual retardation
	27	M	NR	46	58	230/110	NR
Yan et al. [40]	28	M	NR	29	33	240/152	Headache, dizziness, blurred vision, hypertensive heart disease, renal insufficiency
Guo et al. [41]	29	F	NR	29	33	200/120	Acute nocturnal chest pain
	30	F	NR	40	57	140/85 **	NR
Fang et al. [42]	31	M	NR	36	NR	180/120	Type 2 diabetes, OSA, hyperlipidemia, hyperuricemia, hyperinsulinemia, cardiac structural abnormalities

Note: * Patient 14 concurrently carries the chimeric CYP11B1/CYP11B2 fusion gene characteristic of FH-I; ** measured while on nifedipine. Abbreviations: Pt No., Patient Number; M, Male; F, Female; HT, Hypertension; PA, Primary Aldosteronism; BP, Blood Pressure; SBP, Systolic Blood Pressure; DBP, Diastolic Blood Pressure; POTS, Postural Orthostatic Tachycardia Syndrome; OSA, Obstructive Sleep Apnea; NR, Not Reported.

### 3.3. Laboratory Data

Comprehensive biochemical evaluation of the primary cohort (n = 31), including baseline aldosterone and renin profiles, serum potassium levels, and dynamic confirmatory testing (e.g., SIT, CCT), revealed a broad spectrum of mineralocorticoid dysregulation, reflecting substantial quantitative heterogeneity encompassing both classic and atypical biochemical profiles (Table 3).

Potassium levels at the time of diagnostic evaluation were reported as serum concentrations for the majority of the cohort (n = 24), while plasma concentrations were measured in a smaller subset (n = 6, Patients 7–12). Because serum potassium concentrations are generally higher than plasma levels [44], these parameters were analyzed separately. Among serum measurements, potassium ranged from profound hypokalemia (1.52 mmol/L) to upper normal values (4.1 mmol/L), with a median of 3.25 mmol/L (mean: 3.07 mmol/L). In the subgroup evaluated with plasma potassium, values ranged from 1.7 to 3.9 mmol/L, with a median of 2.8 mmol/L (mean: 2.77 mmol/L). While the majority of the overall cohort (21 out of 30 patients with available data, 70%) presented with hypokalemia (defined based on the original authors’ diagnoses or local laboratory reference ranges), a substantial proportion (30%) exhibited normokalemic HT.

Aldosterone levels were available for all 31 patients. While the calculated median PAC was 24.8 ng/dL (mean: 34.5 ng/dL) and values reached extreme levels up to 140.2 ng/dL, it is critical to note that a significant subset of patients (e.g., Patients 14, 15, 16, 17 and 21) presented with PAC values strictly within the normal laboratory reference range [36,37,39]. In accordance, indices of renin secretion, measured either as PRA or PRC, were generally profoundly suppressed across the cohort (e.g., PRA frequently <0.2 ng/mL/h or PRC < 1 mU/L).

Confirmatory dynamic testing, predominantly SIT or CCT, was documented in 11 of 31 patients. Notably, in some cases (Patients 13, 28), the SIT cannot be done due to complications or contraindications [35,40]. In all assessed cases, aldosterone levels failed to suppress below the established diagnostic thresholds (or demonstrated a paradoxical increase, as seen in Patient 19) [39]. Valid PAC values obtained after confirmatory testing ranged from 10.8 ng/dL to as high as 39.4 ng/dL.

**Table 3:** Laboratory findings.

Study [Ref.]	Pt No.	Serum K^+^ [mmol/L]	PAC [ng/dL]	PRA [ng/mL/h]	PRC	ARR	SIT/CCT [ng/dL]
Scholl et al. [19]	1	3.8 ↔	20.0 ↑	<0.10 ↓	NR	>200 ↑ (PRA)	NR
	2	3.1 ↓	66.0 ↑	0.20 ↓	NR	330.0 ↑ (PRA)	NR
	3	3.5 ↔	22.0 ↑	NR	3.0 ↓ [mU/L]	7.3 ↑ (PRC)	NR
	4	3.6 ↔	20.0 ↑	<0.20 ↓	NR	>100 ↑ (PRA)	NR
	5	3.7 ↔	40.0 ↑	<0.70 ↓	NR	>57 ↑ (PRA)	NR
	6	4.1 ↔	87.2 ↑	<0.60 ↓	NR	>145 ↑ (PRA)	NR
Daniil et al. [21]	7	1.7 ↓ Plasma K^+^	119.7 ↑	NR	<1.0 ↓ [mU/L]	22.5 ↑ (PRC)	NR
	8	2.7 ↓ Plasma K^+^	34.8 ↑	NR	3.7 ↓ [mU/L]	7.0 ↑ (PRC)	NR
	9	2.9 ↓ Plasma K^+^	29.0 ↑	NR	2.6 ↓ [mU/L]	5.8 ↑ (PRC)	NR
	10	3.9 ↔ Plasma K^+^	27.2 ↑	NR	3.0 ↓ [mU/L]	5.4 ↑ (PRC)	NR
	11	2.9 ↓ Plasma K^+^	24.8 ↑	NR	<1.0 ↓ [mU/L]	5.0 ↑ (PRC)	NR
	12	2.5 ↓ Plasma K^+^	22.4 ↑	NR	1.5 ↓ [mU/L]	4.5 ↑ (PRC)	NR
Wulczyn et al. [35]	13	3.3 ↓	38.0 ↑	0.17 ↓	NR	223.5 ↑ (PRA)	Aborted
Liu et al. [36]	14 *	3.7 ↔	17.7 ↔	NR	2.3 ↓ [mU/L]	7.7 ↑ (PRC)	17% ↑ (24.9 → 20.7) (CCT)
Zhang et al. [37]	15	3.4 ↓	25.9 ↔	NR	1.1 ↓ [ng/L]	23.5 ↑ (PRC)	12.1 ↑ (SIT)
	16	3.5 ↔	17.9 ↔	NR	11.6 ↓ [ng/L]	1.5 ↔ (PRC)	10.8 ↑ (SIT)
	17	2.4 ↓	20.1 ↔	NR	5.2 ↓ [ng/L]	3.8 ↑ (PRC)	11.1 ↑ (SIT)
Hasini et al. [38]	18	1.9 ↓	24.0 ↑	0.20 ↓	NR	120.2 ↑ (PRA)	NR
Zhu et al. [39]	19	2.7 ↓	16.2 ↑	NR	2.05 ↓ [mU/L]	7.9 ↑ (PRC)	−23.6% ↑ (13.4 → 16.6) CCT)
	20	NR	17.4 ↑	NR	2.05 ↓ [mU/L]	8.5 ↑ (PRC)	NR
	21	2.7 ↓	12.8 ↔	NR	3.44 ↓ [mU/L]	3.7 ↑ (PRC)	NR
	22	3.2 ↓	17.6 ↑	NR	2.05 ↓ [mU/L]	8.6 ↑ (PRC)	NR
Li et al. [34]	23	2.0 ↓	140.2 ↑	<0.20 ↓	NR	>701 ↑ (PRA)	38.3 ↑ (SIT)
	24	2.9 ↓	61.1 ↑	1.84 ↔	NR	33.2 ↑ (PRA)	39.4 ↑ (SIT)
	25	2.9 ↓	35.5 ↑	0.35 ↓	NR	101.4 ↑ (PRA)	26.2 ↑ (SIT)
	26	3.3 ↓	30.8 ↑	<0.20 ↓	NR	162.1 ↑ (PRA)	15.2 ↑ (SIT)
	27	1.5 ↓	21.2 ↑	<0.20 ↓	NR	>106 ↑ (PRA)	11.8 ↑ (SIT)
Yan et al. [40]	28	2.8 ↓	43.0 ↑	NR	23.0 ↔ [ng/L]	1.9 ↔ (PRC) **	Cannot perform (Renal insufficiency)
Guo et al. [41]	29	2.3 ↓	17.9 ↑	0.20 ↓	NR	89.3 ↑ (PRA)	12.9 ↑ (SIT)
	30	3.4 ↓	23.1 ↑	0.40 ↓	NR	57.6 ↑ (PRA)	9.7 (false result—drug interference)
Fang et al. [42]	31	3.9 ↔	28.7 ↑	3.16 ↔	NR	9.1 ↔ (PRA)	NR

Note: ↑ indicates values above the laboratory reference range (or positive test); ↓ indicates values below the laboratory reference range (or negative test); ↔ indicates values within the normal reference range. For ARR, the specific denominator used for calculation is provided in parentheses directly following the value: (PRA) for Plasma Renin Activity or (PRC) for Plasma Renin Concentration. For the CCT, results are presented as the percentage of aldosterone inhibition (suppression), followed by the absolute PAC values before and after captopril administration (baseline → post-test). * Patient 14 concurrently carries the chimeric CYP11B1/CYP11B2 fusion gene characteristic of FH-I. ** ARR elevated in subsequent testing. Abbreviations: Pt No., Patient Number; PAC, Plasma Aldosterone Concentration; PRA, Plasma Renin Activity; PRC, Plasma Renin Concentration; ARR, Aldosterone-to-Renin Ratio; SIT, Saline Infusion Test; CCT, Captopril Challenge Test; NR, Not Reported.

### 3.4. Adrenal Imaging and Histopathology

Adrenal imaging data (computed tomography (CT), magnetic resonance imaging (MRI), or angiography) were available for all 31 patients in the primary cohort (Table 4). Notably, these studies revealed a remarkably diverse morphological spectrum. A substantial proportion of the cohort (14 patients, 45.2%) presented with radiologically normal adrenal glands, while 3 additional patients (9.7%) exhibited only mild unilateral adrenal enlargement or thickening. Conversely, distinct unilateral morphological changes, such as unilateral nodules or hyperplasia, were identified in 7 patients (22.6%), and bilateral adrenal involvement (nodules or hyperplasia) was observed in the remaining 7 patients (22.6%).

AVS was attempted in 9 patients to assess functional lateralization but could not be completed in one case due to poor renal function. Among the 8 successfully evaluated patients, the AVS outcomes were highly heterogeneous. Notably, 4 patients demonstrated clear functional lateralization (lateralization index > 4.0, ranging from 4.2 to 48.0), while the remaining patients exhibited slight lateralization, nondominant secretion, or bilaterally selective aldosterone production.

When comparing AVS outcomes with the corresponding radiological findings, diverse structural and secretory patterns were observed. Specifically, Patient 8, had two right-sided adrenal nodules (21 mm and 11 mm), while the AVS procedure demonstrated a lateralization of aldosterone hypersecretion to the contralateral, morphologically normal left adrenal gland (lateralization index 48.0) [21]. Similarly, Patient 27 exhibited bilateral nodular hyperplasia on imaging but demonstrated strong unilateral secretion (lateralization index 12.16 L) [34]. Patient 14 presented with morphologically normal adrenal glands on CT yet AVS unmasked active unilateral aldosterone secretion from the non-dominant side [36].

Data regarding adrenal histopathology and immunohistochemistry (IHC) were available for only a limited subset of patients who underwent adrenalectomy. In Patient 3, despite a macroscopically normal appearance, histology revealed striking microscopic hyperplasia of the ZG (spanning ~30 cell layers compared to the typical few layers), with micronodular invasion of the capsule and robust CaV3.2 channel expression [19]. In Patient 12, the resected adrenal contained an 8 mm adenoma demonstrating a predominance of ZG-like cells [21]. In a recent study, resected adrenal tumors from two patients (Patients 25 and 26) demonstrated positive CYP11B2 (aldosterone synthase) immunostaining, confirming autonomous aldosterone production within the adenomas, with no underlying somatic mutations detected in the tumor tissue [34]. In contrast, *CYP11B2* immunoreactivity was absent in Patient 27, likely due to a massive hematoma in the resected specimen. Finally, standard hematoxylin-eosin (HE) histopathological examination confirmed adrenocortical adenomas in Patient 29 without further IHC details [41].

**Table 4:** Adrenal imaging findings and functional lateralization via AVS.

Study [Ref]	Pt No.	Adrenal Imaging Findings	Adenoma Size [mm]	AVS Lateralization Index
Scholl et al. [19]	1	No abnormalities (MRI, CT, angiogram)	NR	NR
	2	No abnormalities (CT)	NR	NR
	3	Mild left adrenal enlargement (CT)	NR	4.20 L
	4	No abnormalities (USG, angiogram)	NR	NR
	5	No abnormalities (CT, angiogram)	NR	NR
	6	No abnormalities (angiogram)	NR	NR
Daniil et al. [21]	7	No abnormalities (CT)	NR	NR
	8	Bilateral nodules (CT)	21 R, 11 R	48 L
	9	No abnormalities (CT)	NR	NR
	10	No abnormalities (CT)	NR	NR
	11	Left adrenal hyperplasia (CT)	NR	NR
	12	Left adrenal nodule (CT)	8 L	NR
Wulczyn et al. [35]	13	No abnormalities (MRI)	NR	NR
Liu et al. [36]	14 *	No abnormalities (CT)	NR	Aldosterone secretion from non-dominant side
Zhang et al. [37]	15	No abnormalities (CT)	NR	NR
	16	Mild left adrenal enlargement (CT)	NR	NR
	17	Mild left adrenal diffuse thickening (CT)	NR	NR
Hasini et al. [38]	18	No abnormalities (CT)	NR	NR
Zhu et al. [39]	19	Left adrenal hyperplasia (CT)	6 L	NR
	20	Bilateral adrenal hyperplasia (CT)	NR	NR
	21	Left adrenal hyperplasia (CT)	NR	NR
	22	No abnormalities (CT)	NR	NR
Li et al. [34]	23	Bilateral adrenal nodular hyperplasia (CT)	NR	1.53
	24	Bilateral adrenal nodular hyperplasia (CT)	12.0 R, 15.3 L	2.45 L
	25	Left adrenal nodule (CT)	15 L	6.13 L
	26	Left adrenal nodule (CT)	14 L	NR
	27	Bilateral nodular hyperplasia (CT)	NR	12.16 L
Yan et al. [40]	28	Mild left adrenal hyperplasia (CT)	NR	Cannot perform due to poor renal function
Guo et al. [41]	29	Bilateral adrenal nodules (CT)	NR	1.60 R
	30	Right adrenal nodule, left adrenal hyperplasia (CT)	NR	Not performed
Fang et al. [42]	31	No abnormalities (MRI)	NR	NR

Note: * Patient 14 concurrently carries the chimeric CYP11B1/CYP11B2 fusion gene characteristic of familial hyperaldosteronism type I (FH-I). Abbreviations: Pt No., Patient Number; CT, Computed Tomography; MRI, Magnetic Resonance Imaging; USG, Ultrasonography; AVS, Adrenal Venous Sampling; L, Left; R, Right; NR, Not Reported.

### 3.5. Treatment and Clinical Outcomes

Long-term treatment strategies and clinical outcomes were available for 27 out of the 31 patients in the primary cohort, as 4 patients (Patients 8, 9, 10, and 11) were lost to follow-up shortly after diagnosis or initial intervention [21]. The management approaches varied significantly across the cohort, encompassing both targeted pharmacological therapies and surgical interventions, reflecting the clinical challenges in managing this specific genetic subtype (Table 5).

**Table 5:** Treatment modalities and clinical outcomes.

Study [Ref.]	Pt No.	Surgical Treatment	Final Pharmacological Treatment	BP After Treatment [mmHg]	Laboratory Parameters After Treatment
Scholl et al. [19]	1	None	Eplerenone, Amlodipine, Chlorthalidone	Well controlled	NR
	2	None	Hydrochlorothiazide, Lisinopril	NR	NR
	3	Left adrenalectomy	Hydrochlorothiazide, Lisinopril, Metoprolol	138/87	NR
	4	None	Lisinopril, Amlodipine	118/74	Normal K^+^, PAC 5.0 ↔ [ng/dL], PRA 0.10 ↓ [ng/mL/h], ARR 50.0 ↑ (PRA)
	5	None	Atenolol, Hydrochlorothiazide, Lisinopril, Minoxidil, Potassium chloride	124/65	K^+^ 3.7 ↔ [mmol/L]
	6	None	Enalapril, Spironolactone, Propranolol	NR	NR
Daniil et al. [21]	7	None	Spironolactone	Well controlled	NR
	8	Left adrenalectomy	NR (Lost to follow-up)	Improvement of HT	NR
	9	None	NR (Lost to follow-up)	NR	NR
	10	None	NR (Lost to follow-up)	NR	NR
	11	None	NR (Lost to follow-up)	NR	NR
	12	Left adrenalectomy	None	121/76	PAC 9.7 ↔ [ng/dL], PRC 12.9 ↔ [mU/L], ARR 0.8 ↔ (PRC)
Wulczyn et al. [35]	13	None	Spironolactone, Amiloride	NR	Modestly improved K^+^
Liu et al. [36]	14 *	None	Eplerenone, Amlodipine	115/73	NR
Zhang et al. [37]	15	None	Eplerenone, Nifedipine	Controlled smoothly	Normal K^+^, Normal renal functions
	16	None	Eplerenone, Nifedipine	Controlled smoothly	Normal K^+^, Normal renal functions
	17	None	Eplerenone, Nifedipine	Controlled smoothly	Normal K^+^, Normal renal functions
Hasini et al. [38]	18	None	Spironolactone (temporary), Potassium supplements (temporary)	Normal	Normal K^+^
Zhu et al. [39]	19	None	Spironolactone, Nifedipine	125/85	Normal K^+^
	20	None	Irbesartan, Nifedipine, Metoprolol	130/80	NR
	21	None	Spironolactone, Nifedipine	130/80	NR
	22	None	Spironolactone, Nifedipine	130/80	NR
Li et al. [34]	23	None	Irbesartan, Nifedipine, Potassium chloride	NR	NR
	24	None	Spironolactone, Nifedipine	130–140/80–90	K^+^ 3.8 ↔ [mmol/L]
	25	Left adrenalectomy	None	119/77	K^+^ 3.8 ↔ [mmol/L], PAC 20.9 ↑ [ng/dL], PRA 4.84 ↔ [ng/mL/h], ARR 4.3 ↔ (PRA)
	26	Left adrenalectomy	Valsartan, Potassium supplements	Controlled	NR
	27	Left adrenalectomy	Bisoprolol, Amlodipine	138/81	K^+^ 3.4 ↓ [mmol/L], PAC 8.0 ↔ [ng/dL], PRA 2.05 ↔ [ng/mL/h], ARR 3.9 ↔ (PRA)
Yan et al. [40]	28	None	Spironolactone, Calcium channel blockers, Beta-blockers, Diuretics	130–150/80–100	K^+^ 4.0 ↔ [mmol/L], PAC 99.9 ↑ [ng/dL], PRC 9.2 ↔ [ng/L], ARR 10.9 ↑ (PRC)
Guo et al. [41]	29	Right adrenalectomy	Eplerenone, Benidipine	Optimal	Normal K^+^
	30	None	Spironolactone, Nifedipine	Clinically stable	Normal K^+^
Fang et al. [42]	31	None	Nifedipine, Sacubitril/valsartan	125/83	Normal K^+^

Note: ↑ indicates values above the laboratory reference range; ↓ indicates values below the laboratory reference range; ↔ indicates values within the normal reference range. For (ARR, the specific denominator used for calculation is provided in parentheses directly following the value: (PRA) for Plasma Renin Activity or (PRC) for Plasma Renin Concentration. * Patient 14 concurrently carries the chimeric CYP11B1/CYP11B2 fusion gene characteristic of FH-I. Abbreviations: Pt No., Patient Number; BP, Blood Pressure; HT, Hypertension; K^+^, Potassium; PAC, Plasma Aldosterone Concentration; PRA, Plasma Renin Activity; PRC, Plasma Renin Concentration; ARR, Aldosterone-to-Renin Ratio; NR, Not Reported.

#### 3.5.1. Pharmacological Treatment

The majority of the cohort was managed medically. MRAs, including spironolactone and eplerenone, formed the baseline of pharmacological therapy, effectively improving BP control and normalizing serum potassium levels in most cases. However, a substantial number of patients (e.g., Patients 15, 16, and 17) required a transition to eplerenone [37].

Notably, CCBs, predominantly amlodipine, nifedipine, benidipine, and isradipine, were frequently and successfully utilized alongside MRAs. Despite targeted therapies, achieving optimal BP targets often proved exceptionally challenging, necessitating complex multi-drug regimens. A significant portion of the cohort required combinations involving MRAs, CCBs, angiotensin-converting enzyme (ACE) inhibitors, angiotensin II receptor blockers (ARBs), beta-blockers, and thiazide or thiazide-like diuretics (such as hydrochlorothiazide and chlorthalidone).

The diverse pharmacological approaches also highlighted several unique clinical trajectories within the cohort. For instance, Patient 31 successfully optimized his BP using a modern combination of nifedipine and sacubitril/valsartan, supported by lifestyle modifications including the Dietary Approaches to Stopping Hypertension (DASH) diet and aerobic exercise [42]. Conversely, some cases demonstrated extreme resistance to standard medical therapy. Patient 13, despite being treated with both spironolactone and amiloride, continued to suffer from severe weakness and required monthly intravenous potassium infusions for symptomatic relief [35].

#### 3.5.2. Surgical Interventions and Outcomes

Conversely, the remaining 4 patients (Patients 3, 26, 27, and 29) experienced persistent or rapidly recurrent HT and biochemical imbalances following the removal of the adrenal gland. To maintain BP control and normal clinical parameters, these patients required the postoperative reintroduction of targeted antihypertensive polypharmacy [19,34]. Specifically, Patient 29 underwent a right adrenalectomy but experienced persistent hypokalemia and HT, ultimately requiring ongoing therapy with eplerenone and benidipine. Strikingly, subsequent imaging in this patient revealed the de novo development of a contralateral adrenal adenoma [41]. Patient 27 required postoperative treatment with bisoprolol and amlodipine [34], while Patient 3 required a three-drug regimen comprising hydrochlorothiazide, lisinopril and metoprolol [19], and Patient 26 required valsartan and potassium supplements [34].

#### 3.5.3. Clinical Versus Biochemical Remission

A detailed analysis of post-treatment laboratory parameters revealed a discrepancy between clinical BP control and complete biochemical resolution in some patients. For instance, despite apparent clinical remission after unilateral adrenalectomy (normotension without medications), Patient 25 maintained an elevated PAC (20.9 ng/dL) [34]. Similarly, although postoperative polypharmacy controlled BP in Patient 27, mild hypokalemia persisted (3.4 mmol/L) [34].

### 3.6. Genetic Diagnosis

Detailed genetic data were available for all 31 patients comprising the primary cohort. In all cases, suspicion of FH-IV was based on the identification of heterozygous germline variants in the *CACNA1H* gene. The inheritance patterns demonstrated substantial variability. Specifically, among the 18 cases where familial inheritance could be assessed, 13 exhibited classic autosomal dominant (AD) transmission, while 5 probands harbored confirmed de novo mutations, appearing as the first affected individuals in their respective families. A detailed genetic summary for the entire cohort, including specific nucleotide changes, is provided in Supplementary Material (Table S2).

Overall, a remarkable genetic heterogeneity was observed, with 17 distinct *CACNA1H* variants identified across the small primary cohort. The vast majority of these (15 variants) were missense mutations resulting in single amino acid substitutions, while 2 were recently discovered intronic variants (c.5888-3C>A and c.5324-19G>A [38,39]).

**Figure 4:**
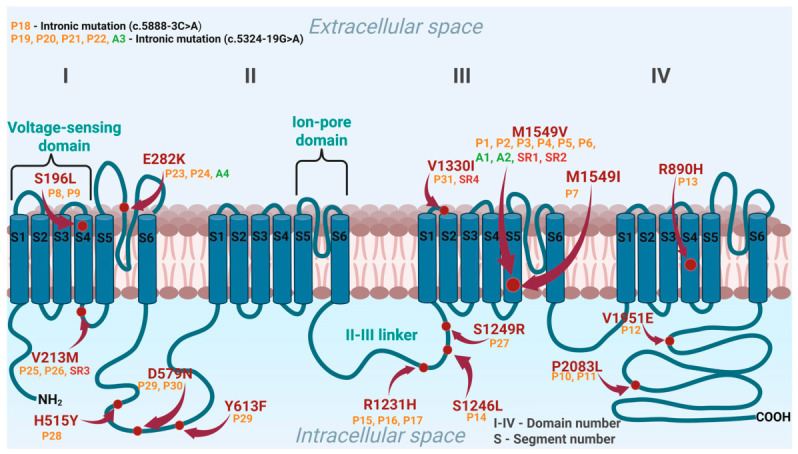
Structural mapping of the identified *CACNA1H* mutation variants. Red dots and corresponding arrows indicate the approximate locations of the identified amino acid substitutions (protein alterations) within the Cav3.2 channel structure. P1–P31 (orange) represent patients included in the primary clinical cohort; A1–A4 (green) denote asymptomatic mutation carriers and SR1–SR4 (red) represent symptomatic relatives excluded from the primary analysis. Notably, P28 exhibits a dual molecular defect, concurrently carrying two distinct variants (p.H515Y and p.Y613F) and P14 concurrently carries the chimeric CYP11B1/CYP11B2 fusion gene. Created in Biorender. Michalski, W. (2026) https://biorender.com/jb07ppj (accessed on 13 March 2026).

Crucially, mapping these 17 variants to the structure of the Cav3.2 channel reveals distinct pathogenetic mechanisms strongly tied to the mutation’s location, which are comprehensively illustrated in Figure 4.

The most frequently reported variant, the missense mutation M1549V (identified in 6 patients of the primary cohort) [19], alongside M1549I, is localized in the critical transmembrane S6 segment of repeat III, which forms the ion-selective pore [21]. Several variants are situated in the intracellular cytoplasmic loops (e.g., Y613F, R1231H, S1249R) [34,37,40] or the C-terminal domain (e.g., P2083L, V1951E) [21].

Adding to the genetic complexity, one patient in the cohort was diagnosed with a dual molecular defect, concurrently carrying a heterozygous *CACNA1H* variant (S1246L) and the chimeric *CYP11B1/CYP11B2* fusion gene characteristic of FH-I [36]. Another patient (Patient 28) presented simultaneously with two distinct missense variants within the *CACNA1H* gene (Y613F and H515Y) [40].

### 3.7. Family History and Asymptomatic Carriers

**Figure 5:**
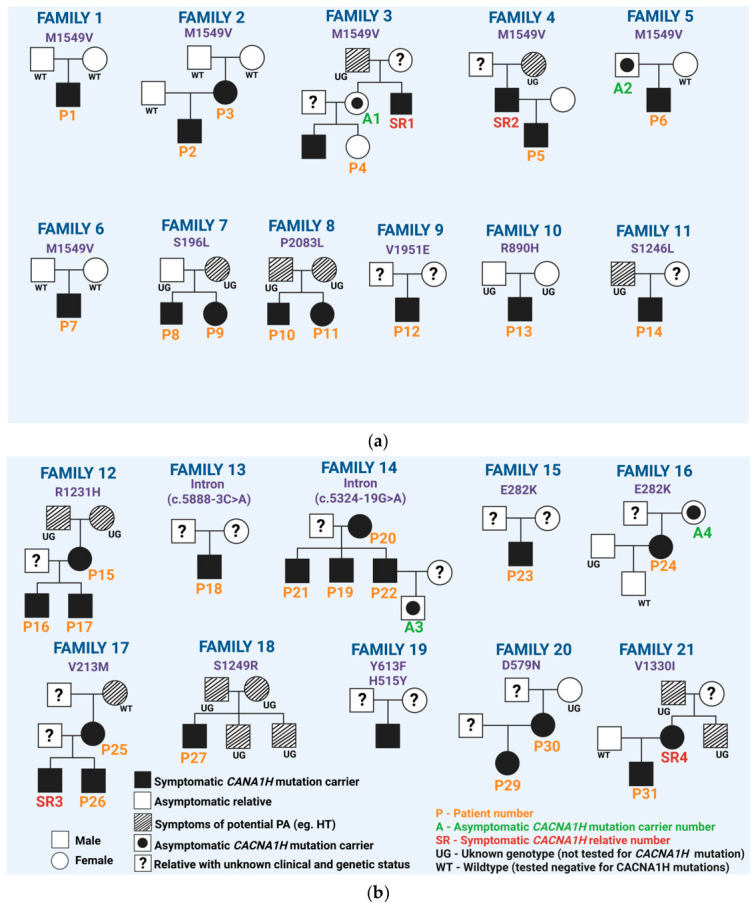
Schematic pedigrees of 21 families included in this review. (**a**) Pedigrees of first 11 families. Created in Biorender. Michalski, W. (2026) https://biorender.com/y67f27t (accessed on 13 March 2026); (**b**) pedigrees of rest 10 families. Created in Biorender. Michalski, W. (2026) https://biorender.com/4ls87b2 (accessed on 13 March 2026). Note: Patient 14 (P14) concurrently carries the chimeric CYP11B1/CYP11B2 fusion gene characteristic of FH-I, and Patient 28 (P28) exhibits a dual molecular defect, concurrently carrying two distinct CACNA1H variants (Y613F and H515Y). Abbreviations: UG—genetic testing not performed); WT—tested negative for germline CACNA1H mutations.

Within the primary cohort of 31 patients, we identified 21 distinct families carrying germline *CACNA1H* mutations. To visually summarize this extensive amount of data, the available inheritance patterns and phenotypic presentations across the cohort are illustrated in a simplified schematic pedigrees (Figure 5a,b), where asymptomatic and symptomatic relatives are distinctly annotated (A1–A4 and SR1–SR4, respectively).

Cascade genetic screening within these 21 families has revealed several first- and second-degree relatives carrying the same pathogenic variants as the probands, 4 of whom were confirmed mutation carriers who remained entirely asymptomatic and normotensive at the time of evaluation (Table 6). The most prominent example is the 12-year-old nephew of Patient 19 (Relative A3), who remains normotensive and normokalaemic despite carrying the familial intronic mutation [39]. Other asymptomatic carriers included adult relatives (e.g., Relatives A1, A2, and A4), whose BP and hormonal profiles remained strictly within normal limits without medical intervention [19,34].

**Table 6:** Clinical, biochemical and genetic characteristics of asymptomatic *CACNA1H* mutation carriers.

Study [Ref.]	Rel. No.	Relative (Linked to Proband)	Age [Years]	BP [mmHg]	Serum (K^+^ [mmol/L]	PAC [ng/dL]	PRA [ng/mL/h]	ARR (PRA)	Variant (cDNA)/Protein Alteration	Inheritance
Scholl et al. [19]	A1	Mother (Patient 4)	NR	116/80	3.7 ↔	8.0 ↔	4.73 ↑	1.7 ↔	c.4645A>G (p.M1549V)	Cannot assess
	A2	Father (Patient 6)	NR	120/82	3.9 ↔	9.3 ↔	0.81 ↔	11.5 ↔	c.4645A>G (p.M1549V)	Cannot assess
Zhu et al. [39]	A3	Nephew (Patient 19)	12	102/65	4.3 ↔	NR	NR	Normal	c.5324-19G>A (Intron)	AD
Li et al. [34]	A4	Mother (Patient 24)	70	119/72	4.2 ↔	5.6 ↔	0.36 ↔	15.6 ↔	c.844G>A (p.E282K)	Cannot assess

Note: ↑ indicates values above the laboratory reference range; ↔ indicates values within the normal reference range.

Conversely, familial screening also identified 4 symptomatic relatives who harbored the familial *CACNA1H* mutation but were excluded from our primary cohort due to incomplete diagnostic biochemical data, despite manifesting with early-onset HT (Table 7). For example, the maternal uncle of Patient 4 (Relative SR1) presented with severe HT (200/100 mmHg) at age 24, and the father of Patient 5 (Relative SR2) developed significant HT (160/120 mmHg) at just 13 years of age. Similarly [19], Relative SR4 (the mother of Patient 31) presented with HT at age 44 and achieved BP control on amlodipine, but lacked a full endocrine evaluation [42]. Finally, the clinical presentation of the son of Patient 25 (Relative SR3) explicitly resulted in the rejection of a formal PA diagnosis by the original investigators; despite developing HT and hypokalemia at age 29 and carrying the V213M variant [34].

**Table 7:** Clinical and genetic characteristics of symptomatic *CACNA1H* mutation carriers not included in the primary cohort.

Study [Ref.]	Rel. No.	Relative (Linked to Proband)	Age of HT Onset	BP [mmHg]	Serum K^+^ [mmol/L]	Variant (cDNA)/Protein Alteration	Inheritance	Clinical Notes & Additional Data
Scholl et al. [19]	SR1	Maternal Uncle (Patient 4)	24	200/100	3.8 ↔	c.4645A>G (p.M1549V)	Cannot assess	Treated with hydrochlorothiazide; PAC 11.0 * ↔ [ng/dL], PRA 1.2 * ↔ [ng/mL/h], ARR 9.2 * ↔ (PRA)
	SR2	Father (Patient 5)	13	160/120	NR	c.4645A>G (p.M1549V)	Cannot assess	NR
Li et al. [34]	SR3	Son (Patient 25)	29	145/97	3.4 ↓	c.637G>A (p.V213M)	AD	OSA, obesity PAC 12.6 ↔ [ng/dL], PRA 3.6 ↔ [ng/mL/h], ARR 3.5 ↔ (PRA), Left adrenal hyperplasia (CT)
Fang et al. [42]	SR4	Mother (Patient 31)	44	131/71 **	Normal **	c.3988G>A (p.V1330I)	Cannot assess	Treated with amlodipine.

**Note:** ↓ indicates values below the laboratory reference range; ↔ indicates values within the normal reference range. * Measured while on hydrochlorothiazide. ** Measured while on amlodipine. Abbreviations: Rel. No., Relative Number; SR, Symptomatic Relative; HT, Hypertension; BP, Blood Pressure; K^+^, Potassium; PAC, Plasma Aldosterone Concentration; PRA, Plasma Renin Activity; ARR, Aldosterone-to-Renin Ratio; NR, Not Reported.

## 4. Discussion

### 4.1. Phenotypic Heterogeneity and Incomplete Penetrance

One of the most striking observations emerging from this review is the substantial phenotypic heterogeneity among carriers of *CACNA1H* mutations. Within the analyzed families, identical genetic variants were associated with markedly different clinical manifestations, ranging from severe early-onset HT with hypokalemia to completely asymptomatic carriers [19,34,39]. The familial cases excluded from the primary quantitative analysis provide particularly illustrative examples of this phenomenon. While several relatives presented with overt manifestations such as early-onset HT or biochemical evidence of mineralocorticoid excess, others remained entirely normotensive with normal biochemical profiles even into advanced age [19,34]. A notable example is the 12-year-old nephew of Patient 19, who carries the familial intronic in vitro variant but remains normotensive and normokalaemic [39]. Similarly, the 70-year-old mother of Patient 24 demonstrated no biochemical evidence of PA despite harboring the same mutation [34]. These findings strongly support the concept of incomplete penetrance in FH-IV. The mechanisms underlying this variability remain incompletely understood. Age-dependent penetrance may partly explain these observations, as aldosterone-producing micronodules and autonomous aldosterone secretion appear to increase with age even in individuals without clinically overt disease [45,46]. Additionally, environmental influences, epigenetic factors, or additional genetic modifiers may modulate the functional impact of *CACNA1H* variants [21,34,41].

### 4.2. Screening Limitations and Genetic Testing

Recent genomic studies have suggested that multiple rare variants affecting adrenal ion channel signaling pathways may interact to influence aldosterone secretion dynamics [47]. Consequently, the true prevalence of FH-IV is likely underestimated, as asymptomatic carriers may remain undiagnosed for extended periods [15]. The present analysis highlights several key diagnostic challenges in FH-IV, including ARR-negative presentations, imaging challenges that may mislead structural diagnostics, and the frequently suboptimal outcomes of surgical interventions such as unilateral adrenalectomy.

The first misleading diagnostic clue may be the aforementioned ARR-negative presentations, as FH-IV can occur without the classical biochemical features of PA [40,42]. Furthermore, a substantial proportion of patients may present with normokalemic HT [19,36,42]. While this lack of classical symptoms can clinically mask the underlying hyperaldosteronism and contribute to diagnostic delays, the biochemical evaluation itself presents an additional diagnostic challenge [16,41,42]. An apparently normal ARR in these patients must be interpreted with extreme caution, as it may represent two distinct scenarios. On one hand, FH-IV may genuinely present with a normal ARR despite normokalemia, as seen in Patient 31 [42]. On the other hand, an apparently normal ARR may simply be a false-negative artifact caused by concurrent hypokalemia (e.g., Patient 28 and Relative SR3, whose potassium levels were 2.8 mmol/L and 3.4 mmol/L, respectively) [34,40]. It is a well-established physiological principle that uncorrected hypokalemia can profoundly suppress aldosterone secretion, thereby biochemically masking the presence of PA [16]. Therefore, failure to correct potassium levels prior to screening remains a major diagnostic limitation. Given these complexities, whether due to genuine normoaldosteronemic phenotypes or artifactual suppression from hypokalemia, reliance solely on classical biochemical screening may lead to missed diagnoses [15,42]. While current clinical guidelines emphasize the ARR as the central screening tool, they increasingly acknowledge that such atypical biochemical presentations may occur in familial forms of the disease [15,16].

The present findings therefore support the growing view that genetic testing should be considered in young patients with severe or familial HT even when ARR screening is inconclusive [41,42]. Advances in NGS have significantly improved the identification of germline variants associated with PA, including *CACNA1H*, *CACNA1D*, *CLCN2*, and *KCNJ5* mutations [14]. Furthermore, broader genomic analyses suggest that the genetic architecture of PA may be more complex than previously recognized, involving multiple rare variants affecting adrenal ion channel signaling pathways [48].

### 4.3. Imaging Diverisity and Discordance

Another important diagnostic challenge identified in this review concerns adrenal imaging, which revealed a remarkably diverse morphological spectrum among the reported cases ranging from completely normal adrenal glands [19,21,35,40] to bilateral nodular hyperplasia [21,34,39,41]. Furthermore, the available data highlight a discordance between radiological findings and actual functional lateralization during AVS in some patients [21,34,36]. As widely recognized in the general clinical management of PA, such radiological-functional discordance is a well-known phenomenon [49]. It is now established that standard cross-sectional imaging frequently fails to reveal obvious adrenal lesions across the entire PA spectrum [7,8,50].

According to the international HISTALDO consensus, PA is often driven by microscopic functional lesions, such as aldosterone-producing micronodules (APMs), which are typically too small to be detected by standard anatomical imaging [8,50]. Consequently, overt PA frequently manifests in morphologically normal-appearing adrenal glands [7]. Therefore, the frequent absence of visible lesions on CT or MRI in these patients is not a unique radiological feature of FH-IV, but rather a predictable consequence of a disease that operates entirely at the molecular level.

These concepts are directly corroborated by histopathological findings from the operated cohort. Tissue analyses reveal a morphological spectrum ranging from microscopic ZG hyperplasia [19], which explains the frequently negative macroscopic imaging, to distinct aldosterone-producing adenomas (APAs) [21,41]. Furthermore, robust *CYP11B2* expression in these APAs without concurrent somatic mutations confirms that germline *CACNA1H* variants alone are sufficient to drive both autonomous aldosterone overproduction and cellular proliferation [34].

### 4.4. Surgical Limitations

The clinical consequences of germline *CACNA1H* mutations are clearly reflected in the suboptimal outcomes of surgical treatment observed across the included studies. In several FH-IV patients who underwent unilateral adrenalectomy, often driven by apparent lateralization in AVS or unilateral imaging findings, complete biochemical and clinical remission was not achieved. Persistent HT and hypokalemia frequently required the postoperative reintroduction of targeted pharmacological polypharmacy [19,34,41].

While it is well-established that specific somatic mutations (e.g., in *KCNJ5*, *CACNA1D*, and *CACNA1H*) can cause focal adrenal adenomas [14,45], the underlying pathogenic mutation in familial forms is present in the germline, meaning both adrenal glands are intrinsically affected [15]. Consequently, structural interventions like unilateral adrenalectomy may only partially reduce aldosterone production. Therefore, unilateral adrenalectomy should be avoided in these patients, rather than being considered an insufficient treatment. Importantly, the preference for medical therapy as a first-line approach over surgery is not exclusive to FH-IV but reflects the broader therapeutic consensus for all channelopathy-related forms of familial PA [15,16].

### 4.5. Pharmacological Management

In our analyzed cohort, a dual pharmacological approach appeared beneficial and was frequently utilized. Combining MRAs with CCBs resulted in smooth BP control and rapid normalization of potassium in Patients 14, 15, 16, 17, 19, 21, 22, and 29 [36,37,39,41]. However, these observations must be interpreted with caution. Given the extremely limited number of reported FH-IV patients and the fact that treatment-resistant HT in PA generally necessitates polypharmacy, which routinely includes MRAs and CCBs, it is difficult to draw definitive conclusions about the specific superiority of this regimen [16,51]. Despite this, many patients still require 3 to 4 antihypertensive medications, underscoring that current treatments remain symptomatic rather than curative [19,40]. Nevertheless, since FH-IV is fundamentally a T-type calcium channelopathy, the choice of the specific CCB subclass used in this polypharmacy is of great pathophysiological importance [19,34]. Supporting this targeted rationale, an in vitro study utilized mibefradil as an experimental tool to demonstrate that specific T-type blockade completely abrogates autonomous aldosterone production in cells expressing mutant *CACNA1H* [52]. Since this non-dihydropyridine agent had already been withdrawn from the market in the late 1990s due to severe *CYP3A4*-related drug interactions, its clinical application for FH-IV is precluded, rendering it strictly a historical “proof-of-concept” [52].

On the other hand, classical dihydropyridine CCBs like nifedipine or amlodipine primarily target L-type channels and do not significantly inhibit T-type channels at therapeutic doses [34]. Despite this, their frequent and effective use in our cohort suggests they are valuable for BP control in these patients. Recent experimental models provide a mechanistic explanation for this efficacy, demonstrating that L-type voltage-gated calcium channels functionally compensate and maintain pathogenic calcium oscillations in ZG cells when T-type channel signaling is altered or inhibited [28]. Furthermore, the latest in vitro functional study supports this concept, showing that targeted L-type blockade with nifedipine directly and dose-dependently inhibits aldosterone overproduction in adrenocortical cells expressing mutant *CACNA1H* variants [34]. However, it should be noted that these findings are strictly based on in vitro models, and it remains currently unknown whether this direct inhibitory effect fully translates to in vivo efficacy [34].

Thus, standard L-type or dual L-/T-type blockade is pathophysiologically justified to suppress this compensatory calcium influx [28]. Notably, the use of benidipine, which has a higher affinity for T-type channels [53], showed promising results in normalizing both BP and potassium [41]. In addition to their adrenal effects, the superior microcirculatory benefits of combined L-/T-type CCBs over conventional L-type blockers likely stem from targeting the abundant T-type calcium channels in the microvasculature [54]. While primarily developed to harness these broader vascular and organ-protective advantages in general HT, ongoing research into novel dual L-/T-type blockers continuously expands the potential therapeutic arsenal for conditions like FH-IV. For instance, toddaculin, a natural plant-derived compound, has recently emerged as one of the promising antihypertensive drug candidates with an additional renoprotective effect [55].

### 4.6. Molecular Challenges and Genotype-Phenotype Correlations

The remarkable genetic heterogeneity of FH-IV represents a major diagnostic challenge. The high ratio of unique variants to the total number of patients indicates that the *CACNA1H* gene lacks a single mutational “hotspot”, being susceptible to pathogenic alterations across its entire sequence. However, while some of identified variants are categorized as pathogenic [19], a substantial proportion remain variants of uncertain significance (VUS) [34,38,56]. This uncertainty reflects the extreme rarity of FH-IV and the limited availability of technically demanding patch-clamp experiments required to demonstrate alterations in channel kinetics [38,40]. Ultimately, ambiguous genetic findings frequently complicate diagnostic decision-making and delay targeted therapeutic strategies.

Emerging evidence suggests that the clinical severity of FH-IV may depend on the specific structural location of mutations within the Cav3.2 channel. Mutations located within the S6 segments of transmembrane domains (e.g., the classical M1549V variant) produce particularly pronounced gain-of-function effects by impairing channel inactivation, frequently causing severe early-onset disease and premature cardiovascular complications [19,21]. Conversely, variants affecting the voltage-sensing S4 segments present a more complex biophysical picture: while S196L alters channel kinetics [21], the R890H variant paradoxically demonstrated a marked decrease in whole-cell current in vitro despite presenting with a clear clinical PA phenotype [35]. Furthermore, mutations affecting intracellular cytoplasmic loops or the C-terminal domain disrupt precise channel regulation by accelerating recovery from inactivation or delaying deactivation, leading to a prolonged, pathological calcium influx [21,34]. Finally, novel intronic mutations (c.5888-3C>A and c.5324-19G>A) highlight potential splicing defects as an additional, underdiagnosed pathogenetic mechanism, suggesting that relying solely on standard whole-exome sequencing (WES) may lead to missed FH-IV diagnoses [38,39]. Although available data remain limited, these findings suggest that genotype–phenotype correlations play an important role in determining disease severity and therapeutic response.

Finally, the familial clustering observed in FH-IV strongly supports the implementation of cascade genetic screening among first-degree relatives. The presence of asymptomatic mutation carriers indicates that FH-IV may remain clinically silent for extended periods [19,34,39]. Early identification of such individuals allows longitudinal monitoring and timely intervention before significant cardiovascular complications develop. Accordingly, European rare disease networks emphasize coordinated genetic screening in hereditary endocrine disorders to optimize clinical outcomes [15].

### 4.7. True Prevalence of FH-IV

Lastly, during our screening we identified multiple conference abstracts describing other, unpublished cases of FH-IV. While our systematic review included exclusively peer-reviewed full-text articles, the necessary exclusion of numerous conference abstracts highlights a substantial underreporting issue. Crucially, these unpublished records do not merely describe asymptomatic incidentalomas, but rather individuals spanning a remarkably wide demographic range, from children as young as 11 years [57,58] to adults up to 75 years of age [59], many of whom suffered from severe, often early-onset, treatment-resistant PA. For instance, excluded cases report profound manifestations such as severe hypokalemia leading to muscle weakness [60], early target-organ damage like concentric left ventricular hypertrophy in adolescents [61], and atypical features including bilateral renal cysts [62]. One record showed remarkable molecular complexities, such as the coexistence of a germline *CACNA1H* variant with a secondary somatic *CTNNB1* mutation in a massive pediatric adenoma [58]. Furthermore, these highly symptomatic patients harbored several entirely novel, potentially pathogenic germline *CACNA1H* variants (e.g., R1823H [57], I153M [60], V1290I [58]), that further expand the channel’s mutational spectrum.

Given the extreme rarity of FH-IV and the remarkably small number of fully documented patients in the literature, we strongly encourage clinicians to publish detailed case reports of any identified individuals. In the context of such an underreported condition, every single case report is an invaluable asset that directly contributes to elucidating the full penetrance, resolving the pathogenicity of VUS, and understanding the clinical diversity and long-term outcomes of *CACNA1H*-related PA. Further studies are needed to develop therapies that act directly on the underlying pathological mechanisms, potentially increasing therapeutic efficacy while minimizing adverse effects.

### 4.8. Summary of the FH-IV Clinical Profile

While a definite diagnosis of FH-IV requires genetic testing, Table 8 summarizes the most prevalent clinical, biochemical, and imaging findings of the cohort, which may serve as a practical clinical clue for early suspicion of the disease.

## 5. Conclusions

FH-IV is a monogenic form of PA characterized by significant phenotypic variability and incomplete penetrance. While classic cases present in infancy, many patients are diagnosed well into adulthood, often following years of unrecognized aldosterone excess despite standard imaging appearing normal. Because FH-IV is a systemic germline disorder, unilateral adrenalectomy is ineffective and often leads to the recurrence of HT and hypokalemia. Consequently, multigene NGS panels should be prioritized in the diagnostic workup for young patients with PA to ensure accurate identification and avoid unnecessary surgeries. While the combination of MRAs and CCBs is widely used in PA, in the context of FH-IV it may have particular therapeutic relevance, with dual L-/T-type CCBs emerging as a potential disease-specific option that addresses both mineralocorticoid excess and the underlying calcium channel dysfunction. Finally, cascade screening of family members is vital for the early detection of asymptomatic mutation carriers, potentially preventing long-term cardiovascular and cerebrovascular complications.

## Figures and Tables

**Table 1:** Inclusion and exclusion criteria applied during the study selection process.

Domain	Inclusion	Exclusion
Population	Patients (pediatric and adult) diagnosed with Familial Hyperaldosteronism Type IV (FH-IV) or *CACNA1H*-related hyperaldosteronism	A. Animal or in vitro studies without linkage to human FH-IV cases B. Patients with other types of familial hyperaldosteronism (FH-I, FH-II, FH-III) without separate FH-IV data C. Sporadic primary aldosteronism without *CACNA1H* involvement D. Studies involving somatic *CACNA1H* mutations (i.e., non-germline variants)
Intervention Exposure	Pharmacological or surgical management of FH-IV, including mineralocorticoid receptor antagonists (MRAs), potassium-sparing diuretics, calcium channel blockers (CCBs), or adrenalectomy	D. Studies focusing on interventions unrelated to FH-IV management E. Basic science studies on calcium channels without clinical or therapeutic relevance
Comparators	Not mandatory	Not mandatory
Outcomes	Clinical outcomes related to blood pressure (BP) control, aldosterone or aldosterone-renin ratio (ARR), electrolyte disturbances, treatment response, or adverse effects	F. Studies without reported clinical outcomes or treatment data
Study designs	Original studies including case reports, case series, observational studies, interventional studies, and clinically relevant functional studies	G. Narrative reviews, editorials, commentaries, guidelines, conference abstracts without original data

Note: Germline mutations are heritable genetic alterations present in all cells of the body (typically identified via peripheral blood sequencing) and are responsible for familial syndromes like FH-IV. Conversely, somatic mutations are acquired, localized exclusively within the adrenal tumor tissue (identified via postoperative tumor sequencing), and are characteristic of sporadic cases of primary aldosteronism (PA).

**Table 8:** Summary of the most prevalent clinical, biochemical, imaging, and therapeutic characteristics of the primary FH-IV cohort (n = 31).

Category	Variable	Prevalent Profile/Findings
Clinical Presentation	Age at HT onset	Frequently early-onset (ranging from 2 months to 48 years)
	Blood pressure	Predominantly severe, treatment-resistant hypertension (often requiring ≥3 medications; peak SBP frequently >160–180 mmHg)
Laboratory Profile	Potassium status *	Hypokalemia: 70% (21/30) Normokalemic HT: 30% (9/30)
	Renin-Aldosterone	Inappropriately elevated PAC, deeply suppressed PRA/PRC, elevated ARR
Adrenal Imaging	Normal/No visible mass	45.2% (14/31)
	Mild enlargement/thickening	9.7% (3/31)
	Unilateral lesions	22.6% (7/31)
	Bilateral lesions	22.6% (7/31)
Genetics	Molecular profile	High heterogeneity (17 distinct *CACNA1H* mutations identified). Most frequent: p.M1549V (S6 segment, repeat III). Intronic mutations present (can be missed by standard WES) Incomplete penetrance (asymptomatic carriers) is observed.
Management & Outcomes	Medical therapy	High requirement for polypharmacy. Regimens typically combine MRAs with other agents, frequently including calcium channel blockers (CCBs).
	Surgical intervention	Unilateral adrenalectomy is generally ineffective for definitive cure and should be avoided.

Note: * One patient in the primary cohort lacked exact potassium data for classification.

## Data Availability

No new data was generated.

## Supplementary Materials

The following supporting information can be downloaded at: https://www.mdpi.com/article/10.3390/jcm15103693/s1, Supplementary S1. Search strategy; Supplementary S2. Supplementary Methods: Detailed Data Extraction and Conversion Rules; Supplementary S3. Evaluation using the JBI critical appraisal checklist for case series; Supplementary S4. Evaluation using the JBI critical appraisal checklist for case reports; Table S1. PRISMA 2020 Checklist; Table S2. Genetic diagnosis of primary cohort.

## Abbreviations

The following abbreviations are used in this manuscript:

FH-IV	Familial hyperaldosteronism type IV
PA	Primary aldosteronism
MRAs	Mineralocorticoid receptor antagonists
CCBs	Calcium channel blockers
NGS	Next-generation sequencing
ZG	Zona glomerulosa
MR	Mineralocorticoid receptor
BP	Blood pressure
HT	Hypertension
IHC	Immunohistochemistry
APAs	Aldosterone producing adenomas
APMs	Aldosterone-producing micronodules
FH	Familial hyperaldosteronism
FH-I	Familial hyperaldosteronism type I
FH-II	Familial hyperaldosteronism type II
FH-III	Familial hyperaldosteronism type III
PASNA	Primary aldosteronism, seizures, and neurologic abnormalities
GRA	Glucocorticoid-remediable aldosteronism
ACTH	Adrenocorticotropic hormone
ARR	Aldosterone to renin ratio
PAC	Plasma aldosterone concentration
PRA	Plasma renin activity
PRC	Plasma renin concentration
SIT	Saline infusion test
CCT	Captopril challenge test
CT	Computed tomography
AVS	Adrenal venous sampling
WES	Whole exome sequencing
PICO	Population, intervention, comparison and outcome
MeSH	Medical subject headings
JBI	Joanna Briggs Institute
NR	Not reported
SBP	Systolic blood pressure
DBP	Diastolic blood pressure
POTS	Postural orthostatic tachycardia syndrome
OSA	Obstructive sleep apnea
MRI	Magnetic resonance imaging
ACE	Angiotensin-converting enzyme
K^+^	Potassium
AD	Autosomal dominant
UG	Unknown genotype
WT	Wild type
SR	Symptomatic relative
VUS	Variant of unknown significance
PRISMA	Preferred Reporting Items for Systematic Reviews and Meta-Analyses
