# Supplementary material for: Familial Hyperaldosteronism Type IV (FH-IV)—Clinical Phenotypes, Genetics and Management of CACNA1H-Related Primary Aldosteronism: A Systematic Review
Source: J Clin Med. 2026 May 11;15(10):3693. doi: 10.3390/jcm15103693 (PMC13207089; doi:10.3390/jcm15103693)
Supplement: Supplementary file 1 [file jcm-15-03693-s001.zip › jcm-4233366-supplementary.pdf]

Supplementary Material

Title: Familial Hyperaldosteronism Type IV (FH-IV)—Clinical Phenotypes, Genetics and Management of CACNA1H-Related Primary Aldosteronism: A Systematic Review

Authors: Wojciech Michalski [1], Igor Jaszczyszyn [2,*], Weronika Bielska [3] and Artur Stolarczyk [1]

1 Department of Orthopaedics and Rehabilitation, Medical and Dentistry Faculty, Medical University

of Warsaw, 02-091 Warsaw, Poland; wojciech.jan.michalski@gmail.com (W.M.);

artur.stolarczyk@wum.edu.pl (A.S.)

2 Doctoral School, Medical University of Warsaw, 02-091 Warsaw, Poland

3 Faculty of Medicine, Medical University of Łódź, 90-419 Łódź, Poland; weroni-ka.bielska@stud.umed.lodz.pl

* **Corresponding author:** igor.jaszczyszyn@wum.edu.pl

Supporting Information - Index

**Supplementary Materials**	
Supplementary S1. Search strategy	*p. 2*
Supplementary S2. Supplementary Methods: Detailed Data Extraction and Conversion Rules	*p. 3*
Supplementary S3. Evaluation using the JBI critical appraisal checklist for case series	*pp. 3-4*
Supplementary S4. Evaluation using the JBI critical appraisal checklist for case reports	*pp. 4-5*
Table S1. PRISMA 2020 Checklist	*pp. 6-10*
Table S2. Genetic diagnosis of primary cohort	*pp. 10-14*

Supplementary S1. Complete search strategy used for the systematic literature review across all included databases. The figure presents the exact Boolean search strings applied to MEDLINE (via PubMed), EMBASE and Web of Science Core Collection.

PubMed:

"Familial Hyperaldosteronism Type IV" OR "Familial hyperaldosteronism type 4" OR "familial hyperaldosteronism IV" OR "FH-IV" OR "FH IV" OR (familial AND hyperaldosteronism AND (type AND (IV OR 4))) OR "CACNA1H" OR (CACNA1H AND (hyperaldosteronism OR "primary aldosteronism" OR aldosteronism)) OR ("Cav3.2" AND (aldosterone OR aldosteronism OR hyperaldosteronism))

Web of science:

ALL=("Familial Hyperaldosteronism Type IV" OR "Familial hyperaldosteronism type 4" OR "familial hyperaldosteronism IV" OR "FH-IV" OR "FH IV" OR (familial AND hyperaldosteronism AND (type AND (IV OR 4))) OR "CACNA1H" OR (CACNA1H AND (hyperaldosteronism OR "primary aldosteronism" OR aldosteronism)) OR ("Cav3.2" AND (aldosterone OR aldosteronism OR hyperaldosteronism)))

Embase:

'familial hyperaldosteronism type iv'/exp OR 'familial hyperaldosteronism type iv' OR 'familial hyperaldosteronism type 4' OR 'familial hyperaldosteronism iv' OR 'fh-iv' OR 'fh iv' OR (familial AND ('hyperaldosteronism'/exp OR hyperaldosteronism) AND type AND (iv OR 4)) OR 'cacna1h' OR (cacna1h AND ('hyperaldosteronism'/exp OR hyperaldosteronism OR 'primary aldosteronism'/exp OR 'primary aldosteronism' OR 'aldosteronism'/exp OR aldosteronism)) OR ('cav3.2' AND ('aldosterone'/exp OR aldosterone OR 'aldosteronism'/exp OR aldosteronism OR 'hyperaldosteronism'/exp OR hyperaldosteronism))

Supplementary S2. Detailed Data Extraction and Conversion Rules.

1. For data collected during the diagnostic workup of PA, BP values represent the highest recorded measurement. Serum or plasma potassium levels correspond to the lowest reported value during the diagnostic process.
2. Due to variability in reported units for PAC (e.g.,, ng/dL, pmol/L, pg/mL, pg/L), all values were converted to ng/dL. Conversions from molar units were performed using the formula: ng/dL = (pmol/L) / 27.7, whereas mass-based units were adjusted using standard metric conversions (e.g., 10 pg/mL = 1 ng/dL). This unit was selected because it was the most frequently reported among the included studies. In cases where both supine and upright aldosterone levels were reported, upright values were recorded, as this measurement was more consistently available when only a single determination was provided.
3. Analogous unit conversions were performed, where possible, for the ARR, PRC, and PRA. As a result, values were standardized to [ng/mL/h] for PRA; and [ng/L] or [mU/L] for PRC. Accordingly, ARR values were expressed as [(ng/dL)/(ng/mL/h)], [(ng/dL)/(mU/L)], or [(ng/dL)/(ng/L)], depending on the specific renin assay utilized.

Supplementary S3. Evaluation using the JBI critical appraisal checklist for case series.

JBI critical appraisal checklist for case series

Study	C1	C2	C3	C4	C5	C6	C7	C8	C9	C10	Total score
Scholl, 2015	Y	Y	Y	Y	Y	Y	U	Y	U	N/A	7
Daniil, 2016	Y	Y	Y	Y	Y	Y	Y	U	U	N/A	7
Wulczyn, 2019	-	-	-	-	-	-	-	-	-	-	-
Liu, 2021	-	-	-	-	-	-	-	-	-	-	-
Zhang, 2024	Y	Y	Y	Y	U	Y	Y	U	Y	N/A	7
Hasini, 2021	-	-	-	-	-	-	-	-	-	-	-
Zhu ZF, 2025	Y	Y	Y	Y	Y	Y	N	U	Y	N/A	7

**Li, 2026**	Y	Y	Y	Y	U	Y	Y	U	Y	N/A	7
**Yan, 2026**	-	-	-	-	-	-	-	-	-	-	-
**Guo, 2026**	Y	Y	Y	Y	U	Y	Y	U	U	N/A	6
**Fang, 2026**	-	-	-	-	-	-	-	-	-	-	-

C1 = Were there clear criteria for inclusion in the case series?
C2 = Was the condition measured in a standard, reliable way for all participants included in the case series?
C3 = Were valid methods used for identification of the condition for all participants included in the case series?
C4 = Did the case series have consecutive inclusion of participants?
C5 = Did the case series have complete inclusion of participants?
C6 = Was there clear reporting of the demographics of the participants in the study?
C7 = Was there clear reporting of clinical information of the participants?
C8 = Were the outcomes or follow up results of cases clearly reported?
C9 = Was there clear reporting of the presenting site(s)/clinic(s) demographic information?
C10 = Was statistical analysis appropriate?
U, unclear; Y, yes; N, no; N/A, not applicable.

Supplementary S4. Evaluation using the JBI critical appraisal checklist for case reports.

JBI critical appraisal checklist for case reports

**Study**	**C1**	**C2**	**C3**	**C4**	**C5**	**C6**	**C7**	**C8**	**Total score**
**Scholl, 2015**	-	-	-	-	-	-	-	-	-
**Daniil, 2016**	-	-	-	-	-	-	-	-	-
**Wulczyn, 2019**	Y	Y	Y	Y	Y	U	U	Y	6
**Liu, 2021**	Y	U	Y	Y	Y	U	U	Y	5

**Zhang, 2024**	-	-	-	-	-	-	-	-	-
**Hasini, 2021**	Y	U	Y	Y	Y	Y	U	Y	6
**Zhu ZF, 2025**	-	-	-	-	-	-	-	-	-
**Li, 2026**	-	-	-	-	-	-	-	-	-
**Yan, 2026**	Y	Y	Y	Y	U	Y	N	Y	6
**Guo, 2026**	-	-	-	-	-	-	-	-	-
**Fang, 2026**	Y	Y	Y	Y	Y	Y	N	Y	7

C1 = Were patient's demographic characteristics clearly described?
C2 = Was the patient's history clearly described and presented as a timeline?
C3 = Was the current clinical condition of the patient on presentation clearly described?
C4 = Were diagnostic tests or methods and the results clearly described?
C5 = Was the intervention(s) or treatment procedure(s) clearly described?
C6 = Was the post-intervention clinical condition clearly described?
C7 = Were adverse events (harms) or unanticipated events identified and described?
C8 = Does the case report provide takeaway lessons?
U, unclear; Y, yes; N, no; N/A, not applicable.

Table S1. PRISMA 2020 Checklist.

Section and Topic	Item #	Checklist item	Location where item is reported
**TITLE**			
Title	1	Identify the report as a systematic review.	p. 1
**ABSTRACT**			
Abstract	2	See the PRISMA 2020 for Abstracts checklist.	p.1
**INTRODUCTION**			
Rationale	3	Describe the rationale for the review in the context of existing knowledge.	p. 5
Objectives	4	Provide an explicit statement of the objective(s) or question(s) the review addresses.	p. 5
**METHODS**			
Eligibility criteria	5	Specify the inclusion and exclusion criteria for the review and how studies were grouped for the syntheses.	pp. 6-7, Table 1
Information sources	6	Specify all databases, registers, websites, organizations, reference lists and other sources searched or consulted to identify studies. Specify the date when each source was last searched or consulted.	p. 7 (2.2. Searching criteria)
Search strategy	7	Present the full search strategies for all databases, registers and websites, including any filters and limits used.	pp. 5-6 (2.2 Searching criteria); Supporting appendix S1

Selection process	8	Specify the methods used to decide whether a study met the inclusion criteria of the review, including how many reviewers screened each record and each report retrieved, whether they worked independently, and if applicable, details of automation tools used in the process.	pp. 5-6 (screening in RAYYAN-QCRI software; two reviewers, independently, with the blind mode enabled; third reviewer consulted for disagreements)
Data collection process	9	Specify the methods used to collect data from reports, including how many reviewers collected data from each report, whether they worked independently, any processes for obtaining or confirming data from study investigators, and if applicable, details of automation tools used in the process.	p. 7 (2.4. Data extraction and collection); Supporting appendix S2
Data items	10a	List and define all outcomes for which data were sought. Specify whether all results that were compatible with each outcome domain in each study were sought (e.g., for all measures, time points, analyses), and if not, the methods used to decide which results to collect.	p. 6-7; Table 1
	10b	List and define all other variables for which data were sought (e.g., participant and intervention characteristics, funding sources). Describe any assumptions made about any missing or unclear information.	p. 6-7; Table 1
Study risk of bias assessment	11	Specify the methods used to assess risk of bias in the included studies, including details of the tool(s) used, how many reviewers assessed each study and whether they worked independently, and if applicable, details of automation tools used in the process.	pp. 7-8 (2.5. Critical appraisal; two reviewers, independently with JBI tools (JBI critical appraisal checklist for cohort studies and the JBI critical appraisal checklist for case series studies)); Supporting appendix S3-S4
Effect measures	12	Specify for each outcome the effect measure(s) (e.g., risk ratio, mean difference) used in the synthesis or presentation of results.	N/A (no effect measures/meta-analysis)

Synthesis methods	13a	Describe the processes used to decide which studies were eligible for each synthesis (e.g., tabulating the study intervention characteristics and comparing against the planned groups for each synthesis (item #5)).	p. 7 (2.4. Data extraction, correction and synthesis)
	13b	Describe any methods required to prepare the data for presentation or synthesis, such as handling of missing summary statistics, or data conversions.	p. 7; Supporting appendix S2
	13c	Describe any methods used to tabulate or visually display results of individual studies and syntheses.	p. 7; Supporting appendix S2
	13d	Describe any methods used to synthesize results and provide a rationale for the choice(s). If meta-analysis was performed, describe the model(s), method(s) to identify the presence and extent of statistical heterogeneity, and software package(s) used.	p. 7; Supporting appendix S2 (narrative synthesis; no meta-analysis)
	13e	Describe any methods used to explore possible causes of heterogeneity among study results (e.g., subgroup analysis, meta-regression).	p. 7; Supporting appendices S3–S4
	13f	Describe any sensitivity analyses conducted to assess robustness of the synthesized results.	N/A
Reporting bias assessment	14	Describe any methods used to assess risk of bias due to missing results in a synthesis (arising from reporting biases).	N/A
Certainty assessment	15	Describe any methods used to assess certainty (or confidence) in the body of evidence for an outcome.	N/A
**RESULTS**			
Study selection	16a	Describe the results of the search and selection process, from the number of records identified in the search to the number of studies included in the review, ideally using a flow diagram.	pp. 8-9; Figure 3
	16b	Cite studies that might appear to meet the inclusion criteria, but which were excluded, and explain why they were excluded.	Not reported

Study characteristics	17	Cite each included study and present its characteristics.	p. 8; Table 1
Risk of bias in studies	18	Present assessments of risk of bias for each included study.	p. 7-8; Supporting appendices S3–S4
Results of individual studies	19	For all outcomes, present, for each study: (a) summary statistics for each group (where appropriate) and (b) an effect estimate and its precision (e.g., confidence/credible interval), ideally using structured tables or plots.	pp. 8–26; Tables 2-7; Supporting Table S2
Results of syntheses	20a	For each synthesis, briefly summarize the characteristics and risk of bias among contributing studies.	pp. 8–26 (narrative synthesis)
	20b	Present results of all statistical syntheses conducted. If meta-analysis was done, present for each the summary estimate and its precision (e.g. confidence/credible interval) and measures of statistical heterogeneity. If comparing groups, describe the direction of the effect.	N/A (no statistical synthesis)
	20c	Present results of all investigations of possible causes of heterogeneity among study results.	p. 7-8; Supporting appendices S3–S4
	20d	Present results of all sensitivity analyses conducted to assess the robustness of the synthesized results.	Not reported
Reporting biases	21	Present assessments of risk of bias due to missing results (arising from reporting biases) for each synthesis assessed.	Not reported
Certainty of evidence	22	Present assessments of certainty (or confidence) in the body of evidence for each outcome assessed.	Not reported
**DISCUSSION**			
Discussion	23a	Provide a general interpretation of the results in the context of other evidence.	pp. 26-30
	23b	Discuss any limitations of the evidence included in the review.	pp. 26-30

	23c	Discuss any limitations of the review processes used.	pp. 26-30
	23d	Discuss implications of the results for practice, policy, and future research.	pp. 26-30
**OTHER INFORMATION**			
Registration and protocol	24a	Provide registration information for the review, including register name and registration number, or state that the review was not registered.	p. 1
	24b	Indicate where the review protocol can be accessed, or state that a protocol was not prepared.	p. 1
	24c	Describe and explain any amendments to information provided at registration or in the protocol.	Not reported
Support	25	Describe sources of financial or non-financial support for the review, and the role of the funders or sponsors in the review.	p. 32
Competing interests	26	Declare any competing interests of review authors.	p. 32
Availability of data, code and other materials	27	Report which of the following are publicly available and where they can be found: template data collection forms; data extracted from included studies; data used for all analyses; analytic code; any other materials used in the review.	pp. 31-37; Supporting appendices S1–S4, Supporting Tables S1-S2

Table S2. Genetic diagnosis of primary cohort.

Study [Ref]	Pt No.	Transcript & Exon	Variant (cDNA)	Protein Alteration	Domain	Inheritance
Scholl et al.	1	NM_021098.3, exon 25	c.4645A>G	p.M1549V	Tm S6, repeat III	De novo
	2	NM_021098.3, exon 25	c.4645A>G	p.M1549V	Tm S6, repeat III	AD

Study [Ref]	Pt No.	Transcript & Exon	Variant (cDNA)	Protein Alteration	Domain	Inheritance
Daniil et al.	3	NM_021098.3, exon 25	c.4645A>G	p.M1549V	Tm S6, repeat III	De novo
	4	NM_021098.3, exon 25	c.4645A>G	p.M1549V	Tm S6, repeat III	AD
	5	NM_021098.3, exon 25	c.4645A>G	p.M1549V	Tm S6, repeat III	AD
	6	NM_021098.3, exon 25	c.4645A>G	p.M1549V	Tm S6, repeat III	AD
	7	NM_021098, exon 25	c.4647G>C	p.M1549I	Tm S6, repeat III	De novo
	8	NM_021098, exon 5	c.587C>T	p.S196L	Tm S4, repeat I	Cannot assess
	9	NM_021098, exon 5	c.587C>T	p.S196L	Tm S4, repeat I	Cannot assess
	10	NM_021098, exon 35	c.6248C>T	p.P2083L	Cytoplasmic, C-terminal	Cannot assess
	11	NM_021098, exon 35	c.6248C>T	p.P2083L	Cytoplasmic, C-terminal	Cannot assess
	12	NM_021098, exon 33	c.5852T>A	p.V1951E	Cytoplasmic, C-terminal	Cannot assess

Study [Ref]	Pt No.	Transcript & Exon	Variant (cDNA)	Protein Alteration	Domain	Inheritance
Wulczyn et al.	13	NM_021098, exon 17	c.2917G>A	p.R890H	Tm S4, repeat II	De novo
Liu et al.	14*	NM_021098.3, exon 17	c.3737C>T	p.S1246L	Cytoplasmic, repeat II-III	De novo
Zhang et al.	15	NM_001005407, exon 17	c.3692G>A	p.R1231H	Cytoplasmic, repeat II-III	Cannot assess
	16	NM_001005407, exon 17	c.3692G>A	p.R1231H	Cytoplasmic, repeat II-III	AD
	17	NM_001005407, exon 17	c.3692G>A	p.R1231H	Cytoplasmic, repeat II-III	AD
Hasini et al.	18	ENST00000348261.1, intron 33	c.5888-3C>A	Intron	Intron	Cannot assess
Zhu et al.	19	NM_021098.3, intron 31	c.5324-19G>A	Intron	Intron	AD
	20	NM_021098.3, intron 31	c.5324-19G>A	Intron	Intron	Cannot assess
	21	NM_021098.3, intron 31	c.5324-19G>A	Intron	Intron	AD

Study [Ref]	Pt No.	Transcript & Exon	Variant (cDNA)	Protein Alteration	Domain	Inheritance
Li et al.	22	NM_021098.3, intron 31	c.5324-19G>A	Intron	Intron	AD
	23	NM_021098.3, exon 7	c.844G>A	p.E282K	Tm S5-S6, repeat I	Cannot assess
	24	NM_021098.3, exon 7	c.844G>A	p.E282K	Tm S5-S6, repeat I	AD
	25	NM_021098.3, exon 5	c.637G>A	p.V213M	Tm S4, repeat I, cytoplasmic end	Cannot assess
	26	NM_021098.3, exon 5	c.637G>A	p.V213M	Tm S4, repeat I, cytoplasmic end	AD
	27	NM_021098.3, exon 18	c.3747C>A	p.S1249R	Cytoplasmic, repeat II-III	Cannot assess
Yan et al.	28	NM_021098.3, exon 9<br>NM_021098.3, exon 9	c.1838A>T<br>c.1543C>T	p.Y613F<br>p.H515Y	Cytoplasmic, repeat I-II<br>Cytoplasmic, repeat I-II	Cannot assess
Guo et al.	29	NM_021098.3, exon 9	c.1735G>A	p.D579N	Cytoplasmic, repeat I-II	AD
	30	NM_021098.3, exon 9	c.1735G>A	p.D579N	Cytoplasmic, repeat I-II	Cannot assess

Study [Ref]	Pt No.	Transcript & Exon	Variant (cDNA)	Protein Alteration	Domain	Inheritance
Fang et al.	31	NM_021098, exon 20	c.3988G>A	p.V1330I	Tm S3, repeat III, extracellular end	AD

Note: *Patient 14 concurrently carries the chimeric CYP11B1/CYP11B2 fusion gene characteristic of FH-1. **Abbreviations:** Pt No., Patient Number; AD, Autosomal Dominant; Tm, Transmembrane; S, Segment.
